# Examining the Effects of Cultural Value Orientations, Emotional Intelligence, and Motivational Orientations: How do LMX Mediation and Gender-Based Moderation Make a Difference?

**DOI:** 10.3389/fpsyg.2020.502903

**Published:** 2020-10-22

**Authors:** Aharon Tziner, Or Shkoler, Erich C. Fein

**Affiliations:** ^1^Peres Academic Center, Rehovot, Israel; ^2^Netanya Academic College, Netanya, Israel; ^3^Independent Researcher, Netanya, Israel; ^4^School of Psychology and Counselling, Centre for Health Research, Institute for Resilient Regions, University of Southern Queensland, Toowoomba, QLD, Australia

**Keywords:** LMX, motivation, counterproductive work behaviors, organizational citizenship behaviors, gender, emotional intelligence, cultural value orientations

## Abstract

We examined the role of leader–member exchange (LMX) as a mediator between individual differences and outcomes across three separate studies with 838 participants. Gender-based moderation was used with the LMX mediation effect. Our results suggest that gender makes a dramatic difference. Specifically, we found that LMX mediation lowered the tendency of counterproductive work behaviors (CWBs) for men. In addition, we found that LMX mediated the effect extrinsic motivation has on extrinsic job satisfaction for women. We trace these differences to a tendency for women to express a more democratic and participative leadership style, which implies a different criterion for leader performance in some situations. We also present suggestions for how the findings of our studies can be extended via organizational practice and future research.

## Introduction

**FIGURE 1:**
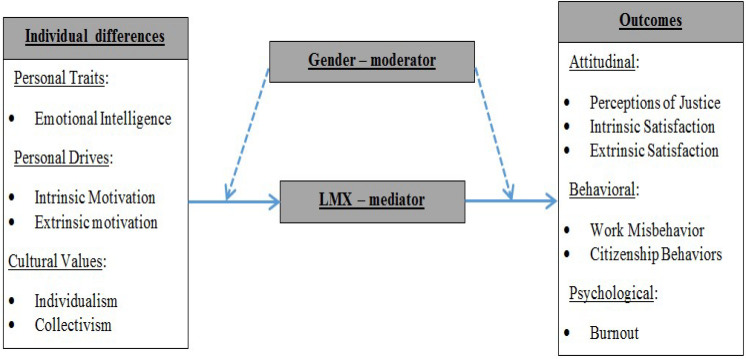
Predictors and outcomes of LMX (as a mediator)—overall model.

Most individuals invest the majority of their waking hours in work activities (Landy and Conte, 2016). Given the extensive time and energy people invest at work, it is of paramount importance to investigate the effects of key workplace factors, such as work-based relationships, that positively influence work outcomes of individuals and organizations. This is even more relevant to the managerial and leadership literature, as many of us work in close proximity or in constant communication with the direct manager at the workplace. So, leadership constructs and processes take a central focus in understanding relationships at work, and this literature leader–member exchange (LMX) theory has been highly successful in explaining critical work outcomes over many decades of research and application (Sharif and Scandura, 2017). Furthermore, adding to the long discussion regarding the effects of environmental vs. individual differences in the work context, in the current paper, we investigate both situational and individual factors engaging LMX in the role of a mediator variable while gender was invoked as a moderator variable. Gender’s effect is particularly important because previous research has indicated that women tend to display different leadership styles than men (e.g., Barsheshet-Picer and Tziner, 2014), that is, democratic and participative styles of leadership to a greater degree than men (Eagly and Johnson, 1990; Appelbaum and Shapiro, 1993; Eagly et al., 2003; van Engen and Willemsen, 2004; Eagly, 2005; Eagly and Carli, 2007). Figure 1 portrays the overall research model.

Employing LMX as a mediator variable, we focus on the dyadic relationships between managers and subordinates as affected by three individual differences—cultural value orientations (CVOs) (individualism vs. collectivism), emotional intelligence, and intrinsic/extrinsic motivational orientations. These individual differences are related to the desired outcomes of organizational citizenship behaviors (OCBs), justice perceptions, and job satisfaction, and the negative outcomes of counterproductive work behaviors (CWBs) and burnout. It is important to emphasize that we contribute to the construct validity evidence for most of the constructs in this paper and that we recognize that individual differences have been scrutinized within the LMX literature. Although the effects of gender-based moderation has been researched, there is still room for further exploration (Zagenczyk et al., 2015), and it is these interactions with gender-based differences on which the current research is intended to shed light. Specifically, we used moderated-mediation models with the same common nexus of mediation and moderation, where we examined the role of LMX as a potential mediator between employees’ individual differences and multiple job-related outcomes along with gender-based moderation.

There is good reason to examine further linkages between gender, LMX, and job-related outcomes. Many scholars suggest that men and women use basically the same mechanisms to create leader behaviors, but there are also subtle, true distinctions (Powell, 1990; Appelbaum and Shapiro, 1993; Eagly, 2005). Specifically, in spite of an amount of gender bias in measuring and defining leadership, there appears to be a basic female leadership style that tends toward a democratic style of relationship enhancement with cooperative and participative leadership behaviors (Eagly and Carli, 2003; Rosener, 2011). Meta-analytic evidence indeed exists to support a gender-specific effect on leadership styles. However, it is worth noting that it dates back to three decades ago. As the state of affairs may have changed, it would be justified to ascertain this effect anew. Consistent with gender-stereotypical expectations about tendencies to lead democratically or autocratically, an early meta-analysis (Eagly and Johnson, 1990) reported that women tend to adopt a more democratic or participative style and a less autocratic or directive style compared to men. This gender difference was shown to occur both within organizations as well as laboratory settings, lending support to the social role theory of sex differences in social behaviors (Eagly and Johnson, 1990). In addition, using research from the 1990s, it was again found that women tend to use more democratic as well as transformational leadership styles compared to men (Eagly et al., 2003; van Engen and Willemsen, 2004).

We posit that much of the basis for how women may present different leader behaviors compared to men can be traced to differences in communication patterns and conflict management strategies and that these patterns and strategies reflect types of processes or exchange elements within LMX. This is in addition to having different expectations in terms of leadership practices. For example, men tend toward a more impersonal style of information exchange during organizational communication, which is in contrast to more relationship-enhancing styles for women in both online and face-to-face communication (Mulac et al., 1998; Sussman and Tyson, 2000; Caruso and Salovey, 2004). Also, with respect to conflict management, women seem to attend to the overall relational context compared to men, and they more readily use cooperative and integrative strategies that work to maximize benefits for all stakeholders and preserve long-term relationships (Brewer et al., 2002).

Based on this strong evidence for an overall democratic, participative tendency for women’s leader behaviors, and the strong evidence base for gender differences in organizational communication and conflict management, we chose to develop three moderated-mediation models, which link LMX mediation and gender-based moderation to various types of attitudinal and behavioral outcomes. We use different models because we wanted to analyze individual differences separately from motivation. However, it is the moderated-mediation pathways that use both LMX mediation and gender-based moderation that serve as the link between the different studies we present in this paper.

At this point, a very worthy point should be made. Although some of the relationships in this study may have been addressed, it is still essential to conduct additional replications. In fact, the late, great mathematician and sociologist Louis Guttman asserted:

But the essence of science is replication: a scientist should always be concerned about what will happen when he or another scientist repeats his experiment. Suppose a regression equation is calculated from one unconditional random sample: what is the variance of prediction made for a new unconditional random sample from the same population on the basis of the previous equation? The answer to this question is unknown; many psychologists are aware of this and therefore do not depend on a single sample but do empirical cross-validation. The same kind of issue, with a different twist, holds for testing hypotheses (Guttman, 1981, p. 25).

## Leader–Member Exchange

Leader–member exchange theory was developed over four decades ago, and it is based on the observation that in dyadic relationships, managers tend to develop and use different relationship and management styles with each of their subordinates (Dansereau et al., 1975; Graen and Cashman, 1975). Different styles of LMX also produce different attitudes in subordinates themselves (Ilies et al., 2007). Capitalizing upon social exchange theory (SET; Blau, 1964) and reciprocity theory (Gouldner, 1960), employees in good relationships with their manager (i.e., high LMX) usually feel obliged to mutually reciprocate according to these relationships (see also Adams, 1965). As such, high-quality LMX results in high levels of trust, respect, and commitment from leaders to subordinates and vice versa. It is important to note that bad relations (i.e., low LMX) with a manager will also tend to result in reciprocal “bad” behavior, and accordingly may eventually lead to CWBs (Ilies et al., 2007; Breevaart et al., 2015; Lebron et al., 2018; Shkoler et al., 2019). However, while LMX’s role as a potential mediator has been investigated (e.g., Sharif and Scandura, 2017), most studies emphasize the prediction of contextual factors, and less is known about the effects of various individual differences as related to performance. In addition, there is even less emphasis on the effects that demographic parameters have on the LMX–performance relationship (Zagenczyk et al., 2015).

## The Moderating Effect of Gender

Leader–member exchange may elicit negative and/or positive outcomes depending on the differentiation the managers make in their relationships with employees. However, this is not true in all cases, and is susceptible to moderating effects (Erdogan and Bauer, 2010). Social role theory suggests that beliefs about gender-appropriate characteristics are societally determined and are translated into differences in behavior between women and men (Eagly, 1987; Eagly and Wood, 2012). Namely, early in life, individuals adapt to the gendered roles that are made available to them by learning and enacting socialized role-related skills (Eagly et al., 2000; Eagly and Wood, 2012). As such, it is possible that the strength of felt gender roles can affect personal predispositions toward other people, especially in key or focal relationships, including those relationships in organizations. It is very plausible that those differences in behavioral predispositions affect many types of work attitudes and evaluations of work states such as job satisfaction and personal preferences and reactions to interactions with leaders. In addition, stereotypes, social categorization, favoritism, and social dominance hierarchies may also have an impact on the behavior of women and men, and these variations in behaviors can lead to differentiating results (McCord et al., 2018).

These gender-based behavioral variations have historically been internalized by the majority of individuals within a society (e.g., McCord et al., 2018) and, ultimately, “through the process of socialization, people come to internalize the gender-typed behaviors that are associated with their own gender role, and they come to expect gender-typed behaviors that conform to the gendered roles of others” (Webster et al., 2018, p. 363). In addition, “these shared expectations for gender-role-congruent behavior produce powerful norms and stereotypes for the behavior and attributes (e.g., sex-typed skills) of women and men (Eagly and Wood, 2012)” (Webster et al., 2018, p. 363). These role-derived differences between genders are socially and culturally cultivated, as are the associated stereotypes with these gender roles. In the end, these role-derived differences between genders and associated stereotypes may elicit different reactions to work situations between women and men (Webster et al., 2018).

## Current Research

In the current paper, we aim to address the gaps mentioned above by investigating traits, drivers, and CVOs as predictors of LMX and different attitudinal, behavioral, and psychological outcomes, across three separate studies, with a specific attention to the role of gender. As such, the emphases and contributions of this research include providing new evidence on the role of LMX as a mediator, taking advantage of an often-disregarded simple demographic parameter—gender—as a moderator, and replicating past research findings in regard to LMX. Accordingly, we aim to show critical differences between males and females, which we believe may be of paramount importance for the understanding of LMX in the organizational and managerial contexts.

To that end, we chose various individual differences as predictors, LMX as the mediator, and several researched outcomes (namely, OCBs, burnout, and CWBs). As stated, we also looked into the gender differences between males and females in this context (see Figure 1 for the overall model), ultimately leading to moderated-mediation models (via multiple-group analyses).

In essence, based on the studies above indicating that women are inclined to develop a more relationship-enhancing style, we expect our studies to illustrate that they are also more likely to develop, *experience*, and *report* higher LMX than men. This perception supposedly impacts in turn upon the dependent variables investigated in this study. It is important to note that, based on extant literature and personal practical experience as consultants with non-academic organizations, of the infinite number of variables at every researcher’s disposal, we have opted for those we deem as dominant in determining work behaviors and work attitudes.

As it is highly difficult to test the overall model in one study, we split the investigation into three different studies, to facilitate survey handling, to indulge participants’ patience, and to advocate parsimonious methodology (see “Discussion” section). Moreover, overly lengthy questionnaires in survey research lead to respondents’ fatigue and lack of interest, thereby affecting the reliability of their responses (Shkoler, 2019). This is corroborated in the literature, as “people can easily quit in the middle of a questionnaire. They are not as likely to complete a long questionnaire… as they would be if talking with a good interviewer” (Phellas et al., 2011, p. 190). We advocated the notion that “questionnaires should take no longer to complete than participants are willing to spend time answering” (Bird, 2009, p. 1312), and, thus, segmented the overall survey into three different questionnaires (i.e., different studies), keeping the number of studies to a minimum as we saw fit.

## Study 1

**FIGURE 2:**
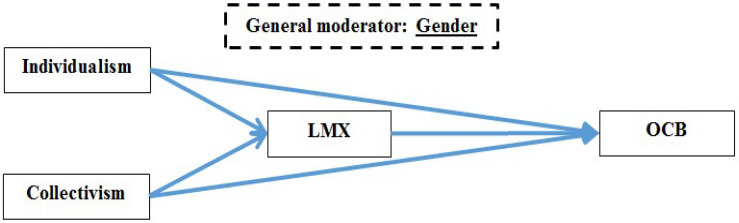
Research model for study 1.

**FIGURE 3:**
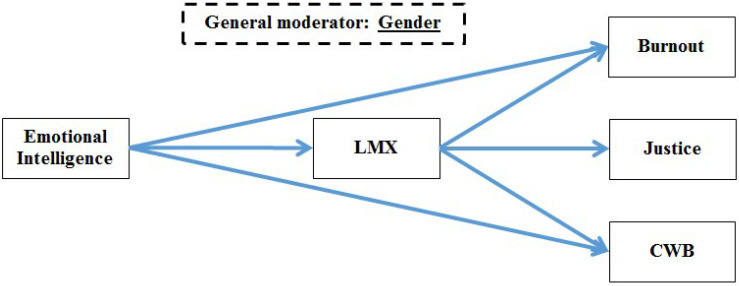
Research model for study 2.

**FIGURE 4:**
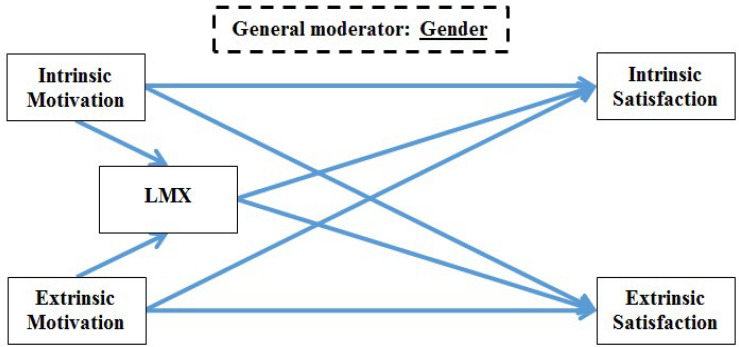
Research model for study 3.

It is imperative to emphasize that the combination of scrutinized variables in each study was based on the vast literature that will be presented hereafter. The decision was not made within a void. However, after all the authors reached consensus, the studies are explored as presented in their respective models (see Figures 2–4 for studies 1, 2, and 3, respectively).

### Cultural Value Orientations

Individualism (IND) and collectivism (COL) can be considered global cultural dimensions that underpin CVOs that may distinguish one individual from another. Our choice of CVOs was based on the fact that they are similar to gender-based behavioral variations, in that they have historically been internalized by the majority of individuals within a society (e.g., McCord et al., 2018) through socialization processes, and because CVOs are similar to gender-based schema due to being oriented toward a major facet of personal identity. The constructs of IND and COL can be applied on the individual (micro-) level as CVOs (see Kirkman et al., 2009), as personal values (e.g., hedonism and altruism)—rather than personality traits (e.g., self-efficacy, extraversion, and emotional intelligence)—just as they are similarly applied to culture as a whole (macro-level). The IND/COL distinction can also be applied to the individual level, employing the definitions of idiocentrism and allocentrism (Triandis, 1989; Chen et al., 2007). *Idiocentric* individuals have IND-based CVOs, and such individuals are typically associated with independence, self-reliance, uniqueness, achievement orientation, and competition (Green et al., 2005; Chen et al., 2007). *Allocentric* individuals have COL-based CVOs, and their behaviors are associated with a sense of duty toward the in-group, interdependence with others, a desire for social harmony and conformity with group norms, and internalizing the group’s goals and values with a high priority (Green et al., 2005; Chen et al., 2007). In other words, the definition of a personal identity is based on group memberships. Whereas collectivists define theirs based on the group, individualists view their personal identity as separate from their memberships in different groups (Hofstede, 1984; Erdogan and Liden, 2006).

Additionally, in relation to LMX, we followed Erdogan and Liden’s (2006) work, and also chose the values of IND/COL instead of the other cultural values, as “individualism/collectivism deals with the relationship orientation of individuals, it influences the factors contributing to the development of interpersonal relationships (Sullivan et al., 2003). In addition, because collectivism defines expectations of individuals from their social system, it may influence how individuals react” (Erdogan and Liden, 2006, p. 4). The authors also based their rationale on social exchange and reciprocity theories (Gouldner, 1960; Blau, 1964) advocated in our current research. Naturally, this notion is cardinal and fundamental to our study.

In spite of the utility of these constructs, in recent years, these two values (individual and cultural) have received both inconsistent and minor attention at the individual level (Allen et al., 2014). Many studies have considered the constructs in the macro-cultural sense as cultural differences (e.g., Allen et al., 2014) and not in the micro-individual sense as individual differences. Those who did research them on the micro-level usually either used them separately or linked them to very few organizational variables (e.g., Özbek et al., 2016). Also, at the individual level, these concepts tend to be used as moderators rather than predictors (Cetin et al., 2015). Most of the cultural-level research on IND and COL is also based on data collected from adolescents or young people (e.g., Lampridis and Papastylianou, 2017), and there is not much evidence regarding the association between prosocial behavior and CVOs. The majority of the studies involving IND and COL assess their relationship indirectly, and the results reported are inconsistent (Lampridis and Papastylianou, 2017, p. 271). Therefore, for the purposes of this study, we chose to examine these specific individual differences as related to prosocial behaviors, such as personal *values*, rather than to personality traits. Additionally, and as will be elaborated further, the study aims at exploring the gender differences in relation to IND and COL.

### Organizational Citizenship Behaviors

Organizational citizenship behaviors are voluntary prosocial behaviors toward the organization or its members, which have a positive impact on effectiveness and efficiency. OCBs are typically seen as outside the formal job description, spontaneous and voluntary behaviors, not apparently or explicitly rewarded, and positive in terms of the organization or group enjoying the behavior (Organ et al., 2006). Such behaviors include helping others with their workload or problem solving, preventing intra-work discord, and working beyond what is required by organizational norms (Organ, 1988). OCBs can stem from job experiences (Zhang et al., 2017) as well as individual differences (Jain, 2016).

When a manager is supportive (e.g., emotional support, trust, information sharing, etc.), employees feel obliged to reciprocate, giving mutual benefit to both sides. However, while LMX’s relationship with OCBs is somewhat clear, the role of cultural values is less so. In spite of the relatively low amount of research in this area, CVOs have indeed been associated with OCBs targeted at an individual in the lower level of organizations (Moorman and Blakely, 1995). However, there is a considerable need to investigate a greater range of psychological processes linking these constructs.

For example, collectivists typically prefer harmony and are inclined to the in-group more closely, and also are much more affected by cohesion and support (from the in-group), as they tend to maintain relationships even when they are not personally advantageous (e.g., Markus and Kitayama, 1991). Thus, assuming they view the manager as part of their in-group, collectivists would likely have a higher affinity to their manager, or reciprocate positive behaviors more readily. On the other hand, because they are achievement orientated and competitive, individualists might see OCBs as a medium to further their work goals and would engage in these behaviors for more personal reasons than collectivists do. In addition, to be consistent with the principle of social harmony, collectivists may try to maintain a good relationship with their manager (Mullen and Skitka, 2009). In contrast, individualists may strive to maintain positive relationships with managers to advance their personal goals or to advance goals that correspond with their own values, like self-actualization, personal growth and development, and individual achievements (Kemmelmeier et al., 2006). Because of the important role of the manager, LMX may also act as a mediator between cultural values and OCBs as the manager provides a focal person at the center of social behaviors. All these expected relationships are articulated via the paths in Figure 2.

#### LMX and OCBs

When managers are supportive of their workers (as characterized, for example, by emotional support, trust, and information sharing), the employees are likely to feel obliged to reciprocate, thus giving mutual benefit to both sides of the manager–worker relationship. One such benefit of supervisors’ support is OCB, which is manifested when employees now recognize that it is worth working beyond their formal job descriptions. They realize that the extra time invested is also one of the few ways they might reciprocate their managers’ support.

#### IND**/**COL and OCBs

While the relationship between LMX and OCBs is clear, the role of cultural values in that relationship is less so. As noted, however, the “collectivists” prefer harmony, are inclined to the in-group more closely, and are much more affected by cohesion and support from the in-group. Thus, they would also have a higher affinity to their manager or reciprocate more readily. On the other hand, individualists, because they are achievement orientated and competitive, might more likely see OCBs as a medium to further their work goals, and would engage in these behaviors for more personal reasons, more so than would the collectivists.

#### IND**/**COL and LMX

For the same reasons, collectivists would try to maintain a strong relationship with their managers (as one of the in-group members; see also Mullen and Skitka, 2009). As indicated, individualists would also do so, but rather to advance those objectives that correspond to their values. These include self-actualization, personal growth and development, and individual achievements (Kemmelmeier et al., 2006). As such, LMX may also act as a mediator between cultural values and OCBs.

#### LMX as a Mediator

We can see that IND/COL values and LMX can be translated into different specific work behaviors (such as OCBs). Additionally, IND/COL might also impact both the *degree* and *quality* of those LMX relationships. Thus, as the culmination of these suppositions, we would expect that personal values of IND/COL would translate to divergent degrees of outcomes (such as in Study 1: LMX and OCBs). That is, LMX may operate as a *mediator* between IND/COL values and OCBs.

#### Gender as a Moderator

The personal values of IND/COL are cultivated in society in much the same manner as gender roles. Thus, as gender affects social outcomes, and personal values lead to varying outcomes in relationships at work, so the interaction between values and gender should promote interesting and differing outcomes and varying relationships with LMX.

Not only might women and men perceive the generic concept of leadership differently, but also, concerning leaders *per se* in the work situation, they may possess disparate attitudes toward their workplace and their respective managers and supervisors, and thus behave differently from one another regarding LMX.

Notably, however, the role of gender, as depicted in Study 1, was as a *general* moderator and not as a specific moderator; that is, not a specific moderation, but a multi-group moderation/analysis consisting of competing models to see if *our* model (for each study, respectively) differs between women and men. The *a priori* prime postulation of the current research is that the models would indeed highlight differences, but, notably, we did not tap into the intricate link-by-link moderation formulation.

It is therefore eminently possible to hypothesize that gender will have a moderating effect in conjunction with LMX, because LMX encapsulates the strength of the relationship between leaders and subordinates. Of course, we recognize that the *specific* effects of gender have been observed in the past (moderations of specific associations, e.g., Beauregard, 2012; Bowling and Burns, 2015; Bell and Khoury, 2016; Webster et al., 2018). However, it needs to be pointed to the absence of studies on the moderating effect of gender on the specific dyadic and directional relationships investigated in this study. Namely, while there is existing research on the moderating role of gender in respect to LMX and outcomes such as OCB (Wang et al., 2017) and personnel decisions (Varma and Stroh, 2001), our concern was based on the moderating roles for gender relative to LMX and the key constructs of CVOs, EI, and intrinsic and extrinsic motivation. Thus, in the current paper, we look at the *overall differences* between genders as associated with LMX (e.g., Khoreva and Tenhiälä, 2016), and this logic, which is articulated in three separate moderated-mediation models, is what unites the separate studies within this paper. Notably, we have chosen not a specific moderation, but a multi-group moderation/analysis with competing models to investigate if our model showcases anticipated differences between women and men (see Figures 1–4, where gender is included as a moderator of *all* paths).

Overall moderation (multi-group moderation/analysis) was chosen over specific moderation for parsimonious reasons. Notably, specific moderation effects would necessitate more variables (e.g., interaction effects) and regression lines in a model (via SEM analyses), thus requiring more degrees of freedom and model complexity, which, in turn, might result in a poorly fitted and defined model.

Furthermore, although culture may be defined as “common patterns of beliefs, assumptions, values, and norms of behavior of human groups (represented by societies, institutions, and organizations)” (Aycan et al., 2000, p. 194), the notion of cultural differences has two related, complementary, but also mutually exclusive aspects. Cultural differences and values are interpreted on the macro-level—the country level of analysis, and the micro-level—the individual level of analysis (Hofstede, 1980, 1991). It is naturally gleaned because macro-CVOs are, eventually, assimilated at other levels (meso- and micro-levels), usually by a top-down mechanism, as “macro socio-cultural contexts influence the acquisition and uses of knowledge in micro-social contexts” (De Abreu, 2000, p. 2), and this top-down process affects “behavioral changes of members in various cultures” (Erez and Gati, 2004, p. 583). In this manner, global culture may affect the national culture, which may impact the organizational culture, which may influence the group culture, which eventually may lead to changes on the individual level (Hofstede, 1980, 1991; Erez and Gati, 2004).

### Study 1 Hypotheses

In light of the above discussion, we arrived at several hypotheses concerning the relationship between IND/COL values with LMX and OCBs, respectively; the relationship between LMX and OCBs; the mediating roles of LMX between IND and OCBs, and COL and OCBs, respectively; and the moderating effect of gender in all these relationships, namely:

H1.1: Individualism has a negative correlation with LMX.
H1.2: Collectivism has a positive correlation with LMX.
H1.3: Individualism has a positive correlation with OCBs.
H1.4: Collectivism has a positive correlation with OCBs.
H1.5: LMX has a positive correlation with OCBs.
H1.6: LMX mediates the relationship between individualism and OCBs.
H1.7: LMX mediates the relationship between collectivism and OCBs.
H1.8: Gender moderates the associations between individualism/collectivism and LMX (as depicted in H1.1–H1.7).

### Method

#### Procedure

The survey research (paper and pencil) was based on the administration of questionnaires by students who participated as research assistants (not as participants). The participation of the respondents in the survey was voluntary. We assured the anonymity and discretion of the participants and the data derived from the research and included a conscious consent question at the beginning of the survey asking for their agreement to participate. No incentives were given whatsoever to the participants for their cooperation (refer to the Ethics Statement section at the end of the paper). In the questionnaire, the participants were assured of our respect for the principle of data confidentiality throughout the entire stages of collection, processing, storage, dissemination, and archiving. Data regarding gender, age, professional experience, education level, and the exercise of a management activity were gathered. Thus, the data become anonymous, making it impossible to identify the respondents. There are no questions in the questionnaire regarding the names, e-mail addresses, telephone numbers, or other personal data of the respondents. In this way, the information was treated responsibly, according to legislation in the field of personal confidentiality of data. All the respondents were employees from various organizations (including high-tech, telemarketing, cellular phone companies, among others). In a way, they could be regarded as convenience investigees.

#### Participants

Cross-sectional data were collected from 245 Israeli workers (all measures were self-reported), 46.5% males (*n* = 114) and 53.5% females (*n* = 131) aged 33–64 years (*M* = 48.22, *SD* = 11.87). Most of them (90.2%) were married, and 9.8% were single; 49.4% held a BA degree and 50.6% held an MA degree or higher. The participants had been working in their jobs between 4 and 38 years (*M* = 21.72, *SD* = 13.38).

#### Measures

*LMX* was gauged by the LMX7 questionnaire (Graen and Uhl-Bien, 1995) consisting of seven Likert-type items; however, items were rescaled to show high LMX (e.g., “extremely effective”) was at the high end of the scale. In the current research, reliability was α = 0.89 (*M* = 3.85, *SD* = 1.02, e.g., “How well does your manager understand your job problems and needs?”).

*OCBs* were gauged by a scale from Williams and Anderson’s (1991) work, consisting of 14 Likert-type items from 1 (“never”) to 6 (“always”). In the current research, reliability was α = 0.76 (*M* = 4.92, *SD* = 0.53, e.g., “Helps others who have heavy workloads”).

The use of full-scale instead of subscales is twofold. First, there are statistical synergies between the subscales, culminating in a “superior” or more efficient full-scale. Namely, the reliabilities and factor loadings of the full-scales are higher than those of their respective subscales [e.g., the first subscale of OCBs (OCB-I; toward individuals) had an alpha of 0.70; the second subscale of OCBs (OCB-O; toward the organization) had an alpha of 0.66]. However, when loading on a single full-scale factor, the alpha received was 0.76, indicating, from a statistical perspective, that it is better in this sample to employ the measure in its full-scale form (as reliabilities are sample-dependent).

Second, the focus of the paper was LMX and gender. We employed full-scales (additionally) to avoid diverting readers’ attention from the primary goal by using multiple subscales and over-complex models.

*Individualism/Collectivism* was gauged by the Individualism and Collectivism Scale (IND-COL; Min Jung et al., 2009), consisting of 10 Likert-type items from 1 (“strongly disagree”) to 6 (“strongly agree”). In the current research, for *individualism*, reliability was α = 0.76 (*M* = 3.16, *SD* = 0.67, e.g., “Acting as an individual is more appealing to me than acting as a member of a group”); for *collectivism*, reliability was α = 0.77 (*M* = 3.61, *SD* = 0.66, e.g., “I will sacrifice my self-interest for the benefit of the group I am in”).

### Results

#### Common-Method Bias

To assess the extent to which inter-correlations among the variables might be an artifact of common method variance (CMV), we employed three tests: (a) the Harman’s single-factor method [a confirmatory factor analysis (CFA) in which all items are simultaneously loaded on one single factor]; (b) a common latent factor method (a CFA in which all items are loaded on both their expected factors and one common latent factor is loaded on each of the items respectively, but are uncorrelated to their respective latent factors); and (c) a CFA without a common latent factor, as suggested by Podsakoff et al. (2003) and advocated in Jawahar et al. (2018). The Harman’s single-factor method accounted only for 30.47% of the explained variance: χ^2^(244) = 1513.77, *p* = 0.000, χ^2^/df = 6.20, CFI = 0.61, NFI = 0.77, GFI = 0.84, SRMR = 0.19, RMSEA (90% CI) = 0.29 (0.12–0.34), *p-close* = 0.000. (We added CIs for the RMSEA, as well as in other places in the entire paper, as per Dilchert’s, 2017, suggestion to include them, when applicable, in empirical research). In addition, the common latent factor accounted only for 27.31% of the explained variance: χ^2^(241) = 925.17, *p* = 0.000, χ^2^/df = 3.83, CFI = 0.78, NFI = 0.83, GFI = 0.88, SRMR = 0.11, RMSEA (90% CI) = 0.15 (0.09–0.21), *p-close* = 0.004. Last, the CFA analysis (without a common latent factor) accounted only for 28.44% of the explained variance: χ^2^(182) = 991.05, *p* = 0.000, χ^2^/df = 5.44, CFI = 0.77, NFI = 0.80, GFI = 0.91, SRMR = 0.10, RMSEA (90% CI) = 0.13 (0.00–0.19), *p-close* = 0.000. As can be seen, the common latent factor method produced better indices and less CMV. While these results do not rule out completely the possibility of same-source bias (i.e., CMV), following Podsakoff et al. (2003), less than 50% (*R*^2^ < 0.50) of the explained variance accounted for by the first emerging factor—in conjunction with the poor model fit for each analysis—indicates that CMV is an unlikely explanation of our investigation’s findings. In addition, we followed the suggestion for correcting CMV via construct-level correction indicated by Tehseen et al. (2017) and discovered that the changes in coefficient strength were very negligible. This observation, again, indicates that our results did not suffer from CMV issues.

**FIGURE 5:**
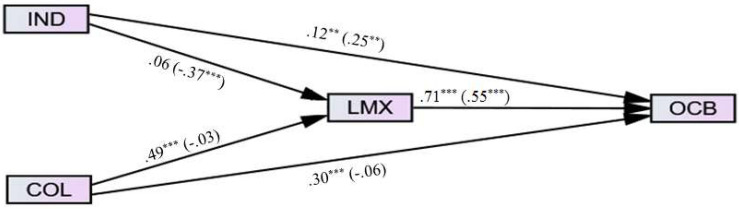
Path diagram for male group (*n* = 114) and female group (*n* = 131; in parenthesis), study 1. *p < 0.05, **p < 0.01, ***p < 0.001. χ^2^(df) = 22.77 (2), *p* = 0.433, χ^2^/df = 11.38, SRMR = 0.02, CFI = 0.93, GFI = 0.96, NFI = 0.93, ECVI = 0.24, RMSEA (90% CI) = 0.20 (0.13–0.28), *p*-close = 0.000.

To test the Study 1 model, we employed SEM with multi-group moderation analyses using the observed (not latent) variables of the research. The path diagrams for the male group and the female group are presented in Figure 5, with coefficients and significance levels (and fit indices). The bivariate correlation matrix is presented in Table 1.

**TABLE 1:** Correlation matrix for males (*n* = 114) and females (*n* = 131; *above* the diagonal) for Study 1.

	1	2	3	4	*M*_*m*_ (*M*_*f*_)	*SD*_*m*_ (*SD*_*f*_)
(1) Collectivism	–	0.40***	−0.36***	0.02	3.20 (3.12)	0.71 (0.62)
(2) Individualism	−0.04	–	−0.18*	−0.05	3.58 (3.63)	0.74 (0.58)
(3) LMX	0.03	0.49***	–	0.47***	3.82 (3.87)	1.16 (0.89)
(4) OCBs	0.13	0.64***	0.86***	–	4.84 (4.98)	0.59 (0.47)

*p < 0.05, ***p < 0.001. An indication of m or f: m, male group; f, female group.

As can be seen in Figure 5, the model’s fit is marginal; not all the indices are adequate (see Byrne, 2010). In terms of mediation effects, not all the mediation conditions were met in each model where the significant effects of (1) predictor → criterion; (2) predictor → mediator; (3) mediator → criterion should be present; and (4) the direct effect should be less than the total effect (Hayes, 2013). Therefore, when testing for the significance of the mediation effect via bootstrapping (see Preacher and Hayes, 2008), we chose only the paths that met *all* the aforementioned mediation conditions. We used the R software package (v. 3.4.1) for employing a recent effect size estimate (kappa-squared = k^2^; Preacher and Kelley, 2011) of the indirect mediation effect with a 95% confidence interval bootstrapping.

This resulted in a standardized indirect effect (collectivism → LMX → OCBs) for *males* of 0.31 (95% CI: 0.23, 0.45; k^2^ = 0.42, *p* = 0.000). The standardized indirect effect (individualism → LMX → OCBs) for *females* was −0.18 (95% CI: −0.47, −0.04; k^2^ = 0.21, *p* = 0.013). Most notable is the gender difference for this effect size between the two models. Also, in contrast to our expectation, collectivism was negatively correlated with LMX, only in the *female* group. This path was non-significant in the *male* group. Table 2 summarizes the results.

**TABLE 2:** Hypotheses summary (Study 1).

Hypotheses	Male group	Female group
H1.1: Individualism has a negative correlation with LMX	Supported	Supported
H1.2: Collectivism has a positive correlation with LMX	Supported	Not supported
H1.3: Individualism has a positive correlation with OCBs	Supported	Not supported
H1.4: Collectivism has a positive correlation with OCBs	Not supported	Not supported^*a*^
H1.5: LMX has a positive correlation with OCBs	Supported	Supported
H1.6: LMX mediates the relationship between individualism and OCBs	Supported	Not supported
H1.7: LMX mediates the relationship between collectivism and OCBs	Not supported	Supported
H1.8: Gender moderates the associations between individualism/collectivism and LMX	Supported	

*^a^Although statistically significant, the (negative) relationship is contrary to the hypothesis.*

## Study 2

### Emotional Intelligence (EI)

Emotions play an important part in the manager–worker relationship because they affect the quality of LMX (Cropanzano et al., 2017). Emotional intelligence (EI) is generally defined as a trait that reflects an awareness of one’s own and other people’s emotions that enables an individual to distinguish between different feelings and to use emotional information to guide thought, behavior, and performance (Boyatzis, 2009; Joseph and Newman, 2010). EI is based in (a) self-awareness, (b) self-management, (c) self-control, (d) adaptability and flexibility, (e) achievement orientation, and (f) a positive point of view (Boyatzis, 2009). Furthermore, the regulation of emotions helps employees to maintain a positive state of mind (Joseph and Newman, 2010; Shkoler and Tziner, 2017). Again, we draw on social role theory and its suggestions that beliefs about gender-appropriate characteristics are societally determined (Eagly, 1987; Eagly and Wood, 2012). In respect to expectations regarding the handling of emotions and according EI, we note that early in life, individuals adapt to the gendered roles that are made available to them by learning and enacting socialized role-related skills, and that such social skills can include changes in the management of emotion (Arnett et al., 2018). As such, it is possible that the strength of felt gender roles can affect personal predispositions toward regulating emotions toward other people and hence could be connected to EI.

### Organizational Justice

Organizational justice (OJ) is the extent to which employees are provided with appropriate, fair, and respectful treatment, information, and resources and rewards (Fein et al., 2013). These perceptions are a product of overall impressions based on a consequence of organizational occurrences and personal evaluations based on specific “organizational components,” such as leaders and co-workers (Hollensbe et al., 2008). Typically, OJ comprises (a) *distributive* justice, (b) *procedural* justice, and (c) *interactional* justice (Fein et al., 2013). However, for parsimonious reasons, in the present study, we investigated the *overall* perception of justice (e.g., Shkoler and Tziner, 2017).

### CWBs and Burnout

In contrast to OCBs, CWBs have received increasing attention on both the academic and the organizational fronts (Ho, 2012; Shkoler and Tziner, 2017; Lebron et al., 2018; Shkoler et al., 2019) due to their significant economic, sociological, and psychological implications (Aubé et al., 2009). CWBs, which may include theft, sabotage, withdrawal, or harassment, are directed at either the organization itself or its members (Welbourne and Sariol, 2017; Bragg and Bowling, 2018). CWBs usually damage organizations in various ways (Robinson, 2008). There are many antecedents to CWBs, such as *individual differences* (Palmer et al., 2017), *job experiences*, *work stressors* (Welbourne and Sariol, 2017), and more. To indicate a negative exemplar, we examined work burnout, which is usually described as a psychological state related to stress over time and is composed of (a) emotional exhaustion, (b) experienced distance from others (depersonalization), and (c) feelings of reduced personal accomplishment/efficacy associated with a variety of negative outcomes (Anthony-McMann et al., 2017). Moreover, burnout may also be affected by *individual differences*, *job experiences*, and *work stressors* (e.g., Nahrgang et al., 2011; Tziner et al., 2018; Rabenu et al., 2019; Tziner et al., 2019a).

A manager’s support, trust, rewards, transparency, and respect are some of a worker’s resources in the job (see Hobfoll, 1989). When the LMX is high, those resources are more frequent and abundant; they might well help to prevent employees from burning out and lead them to perceive their workplace as fairer and more just. Positive relationships with managers assuage workers’ negative experiences and may be reciprocated with good behavior on the employee’s part, giving them less reason to engage in CWBs.

#### EI and Burnout

On the other hand, what the manager cannot provide is EI, which is a *personal* trait. As a highly important personal resource, EI regulates feelings, facilitates the processing and understanding of emotional information, and, when present at high levels, promotes a positive state of mind that may help workers cope, thus decreasing and even preventing burnout.

#### EI and CWBs

Emotions play a crucial role in workplace incivility (e.g., Sears and Humiston, 2015), and emotional regulation is a prime ingredient in “keeping it cool and calm.” This positive state of mind also reduces the possibility that workers will engage in CWBs (for a meta-analysis, see Miao et al., 2017).

#### EI, LMX, and Justice

Because of its beneficial elements, EI may also facilitate efficient judgment of the work context and interpersonal activities. Being self-regulated, positive, and calm ultimately may help employees create a better-quality relationship with the manager (i.e., high LMX) and to perceive the organization in a brighter light (i.e., perceptions of justice). As such, LMX may also act as a mediator.

#### LMX as a Mediator

We can see that EI and LMX can be translated into different work behaviors (i.e., CWBs), attitudes (i.e., OJ perceptions), and even psychological outcomes (i.e., burnout). Additionally, EI might also impact the degree and quality of LMX relations. Thus, as the culmination of previous arguments, LMX may operate as a *mediator* between EI and CWBs, OJ perceptions, and work burnout.

#### Gender as a Moderator

Here, as well, we expect gender differences. Regarding Study 2, it is known that women have higher EI than men (e.g., Mandell and Pherwani, 2003; Brackett et al., 2004), and thus may exhibit different attitudes or behaviors at work.

Figure 3 summarizes the main themes discussed above, according to the following logic. When LMX is high, resources to prevent employees’ burnout are more frequent and/or abundant, potentially leading to a negative association between LMX and burnout. This negative association may also be helpful in perceiving the workplace as fairer and more just, potentially leading to a positive association between LMX and justice perceptions. A strong relationship with the manager might assuage negative experiences, giving less reason to engage in CWBs, as such a relationship would be reciprocated with employees’ good behavior. Managers, however, cannot provide the EI that might facilitate sound judgment of the work context and interpersonal activities that would reduce CWBs. Strong EI promotes self-regulation, positive attitudes, and a calm disposition that promote quality relationships with managers and positive perceptions of justice in the organization. As such, LMX may also act as a mediator between EI and justice, EI and burnout, and EI and CWBs. It is also known that women have higher EI than men (e.g., Mandell and Pherwani, 2003), and thus may be expected to exhibit different attitudes or behaviors at work than their male colleagues, such as the significant moderating role that gender plays in reactions to injustice (Khoreva and Tenhiälä, 2016). Thus, concerning the relationships described in this study, we also expect moderation effects based on gender. These relationships are articulated in the expected paths illustrated in Figure 3 and the hypotheses listed below.

### Study 2 Hypotheses

H2.1: LMX has a positive correlation with organizational justice.
H2.2: LMX has a negative correlation with burnout.
H2.3: LMX has a negative correlation with CWBs.
H2.4: EI has a positive correlation with LMX.
H2.5: EI has a positive correlation with organizational justice.
H2.6: EI has a negative correlation with burnout.
H2.7: EI has a negative correlation with CWBs.
H2.8: LMX mediates the relationship between EI and organizational justice.
H2.9: LMX mediates the relationship between EI and burnout.
H2.10: LMX mediates the relationship between EI and CWBs.
H2.11: Gender moderates the associations between EI, LMX, organizational justice, burnout, and CWBs (as depicted in H2.1–H2.10).

### Method

#### Procedure

The survey research (paper and pencil) was based on the administration of questionnaires by students who participated as research assistants (not as participants). The participation of the respondents in the survey was voluntary. We assured the anonymity and discretion of the participants and the data derived from the research and included a conscious consent question at the beginning of the survey asking for their agreement to participate. No incentives were given whatsoever to the participants for their cooperation (refer to the Ethics Statement section at the end of the paper).

#### Participants

Cross-sectional data were collected from 243 Israeli workers (all measures were self-reported) from various organizations via voluntarily surveys, 48.1% males (*n* = 117) and 51.9% females (*n* = 126) aged between 20 and 60 (*M* = 32.67, *SD* = 8.87). Most of them (83.4%) held a BA degree, and 16.4% held an MA degree or higher. The participants had been working in their jobs between 0 and 48 years (*M* = 6.69, *SD* = 8.17).

#### Measures

*LMX* was gauged by the LMX7 questionnaire (Graen and Uhl-Bien, 1995) consisting of seven Likert-type items; however, each item had a different scale from 1 to 6. In the current research, reliability was α = 0.87 (*M* = 4.15, *SD* = 1.04).

*CWBs* were measured by the Interpersonal and Organizational Deviance Scale (IODS; Bennett and Robinson, 2000), consisting of 19 Likert-type items between 1 (“never”) and 6 (“every day”). In the current study, reliability was α = 0.76 (*M* = 1.67, *SD* = 0.54, e.g., “Taken property from work without permission”).

*Organizational justice* was measured using the Justice Scale (Niehoff and Moorman, 1993), consisting of 20 Likert-type items between 1 (“completely disagree”) and 6 (“completely agree”). In the current study, reliability was α = 0.76 (*M* = 3.95, *SD* = 1.01, e.g., “I consider my workload to be quite fair”).

*Emotional intelligence* was gauged using the Trait Emotional Intelligence Questionnaire—Short Form (TEIQue-SF; Petrides, 2009), consisting of 30 Likert-type items between 1 (“very little”) and 6 (“very much”). In the current study, reliability was α = 0.76 (*M* = 4.47, *SD* = 0.45, e.g., “Expressing my emotions with words is not a problem for me”).

*Burnout* was measured with the Maslach Burnout Inventory (MBI; Maslach and Jackson, 1981), consisting of 22 Likert-type items between 1 (“a few times a year”) and 6 (“every day”). In the current study, reliability was α = 0.95 (*M* = 2.34, *SD* = 0.96, e.g., “I feel emotionally drained from my work”).

### Results

#### Common-Method Bias

In order to assess the extent to which inter-correlations among the variables might be an artifact of CMV, we employed three tests: (a) the Harman’s single-factor method (a CFA in which all items are simultaneously loaded on one single factor), (b) a common latent factor method (a CFA in which all items loaded are on both their expected factors and one common latent factor is loaded on each of the items, respectively, but are uncorrelated to their respective latent factors), and (c) a CFA without a common latent factor, as suggested by Podsakoff et al. (2003) and advocated in Jawahar et al. (2018). The Harman’s single-factor method accounted only for 21.16% of the explained variance: χ^2^(371) = 1296.55, *p* = 0.000, χ^2^/df = 3.49, CFI = 0.70, NFI = 0.75, GFI = 0.89, SRMR = 0.16, RMSEA (90% CI) = 0.15 (0.11–0.20), *p-close* = 0.016. In addition, the latent common method factor analysis accounted only for 20.09% of the explained variance: χ^2^(363) = 1174.62, *p* = 0.000, χ^2^/df = 3.23, CFI = 0.84, NFI = 0.87, GFI = 0.91, SRMR = 0.11, RMSEA (90% CI) = 0.10 (0.04–0.19), *p-close* = 0.024. Last, the CFA analysis (without a common latent factor) accounted only for 21.04% of the explained variance: χ^2^(308) = 1,089.13, *p* = 0.000, χ^2^/df = 3.53, CFI = 0.79, NFI = 0.82, GFI = 0.90, SRMR = 0.09, RMSEA (90% CI) = 0.13 (0.07–0.18), *p-close* = 0.000. As can be seen, the common latent factor method produced better indices and less CMV. However, while these results do not rule out completely the possibility of same-source bias (i.e., CMV), following Podsakoff et al. (2003), less than 50% (*R*^2^ < 0.50) of the explained variance accounted for by the first emerging factor indicates that CMV is an unlikely explanation of our investigation’s findings, in conjunction with the bad model fit for each analysis. In addition, we followed the suggestion for correcting CMV via construct-level correction made by Tehseen et al. (2017) and discovered that the changes in coefficients’ strength were very negligible. This, again, indicates that our results did not suffer from CMV issues.

**FIGURE 6:**
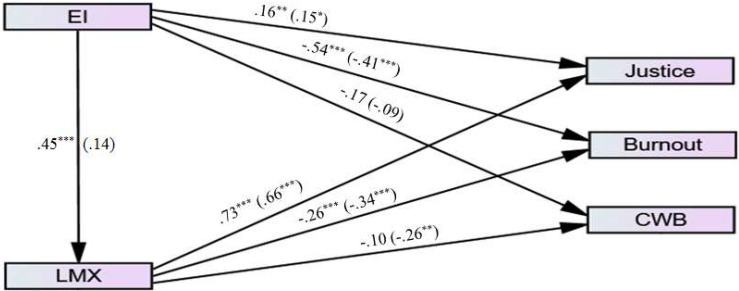
Path diagram for male group (*n* = 117) and female group (*n* = 126; in parenthesis), study 2. *p < 0.05, **p < 0.01, ***p < 0.001. χ^2^(df) = 1.04 (2), *p* = 0.595, χ^2^/df = 0.52, SRMR = 0.01, CFI = 1.00, GFI = 0.99, NFI = 0.99, ECVI = 0.23, RMSEA (90% CI) = 0.00 (0.00–0.11), *p*-close = 0.745.

To test the Study 2 model, we employed SEM with multi-group moderation analyses using the observed (not latent) variables of the research. The path diagrams for the male group and the female group are presented in Figure 6, with the coefficients and their significance levels (and fit indices). The bivariate correlation matrix is presented in Table 3.

**TABLE 3:** Correlation matrix for males (*n* = 117) and females (*n* = 126; *above* the diagonal) for Study 2.

	1	2	3	4	5	*M*_*m*_ (*M*_*f*_)	*SD*_*m*_ (*SD*_*f*_)
(1) EI	–	0.14	−0.45***	0.24***	−0.12	4.53 (4.41)	0.42 (0.47)
(2) LMX	0.45***	–	−0.39***	0.68***	−0.27**	4.26 (4.06)	0.99 (1.08)
(3) Burnout	−0.65***	−0.50***	–	−0.52***	0.34***	2.18 (2.48)	0.89 (0.98)
(4) Justice	0.49***	0.80***	−0.61***	–	−0.22**	4.14 (3.79)	0.95 (1.05)
(5) CWBs	−0.21*	−0.18*	0.32***	−0.21**	–	1.58 (1.51)	0.56 (0.51)

*p < 0.05, **p < 0.01, ***p < 0.001. An indication of m or f: m, male group; f, female group.

As can be seen in Figure 6, the model’s fit is strong (see Byrne, 2010). We followed our analyses and processes, as in Study 1, and we used kappa-squared to test the indirect mediation effects with a 95% bootstrapped CI.

This resulted in a standardized indirect effect (EI → LMX → OJ) for *males* of 0.29 (95% CI: 0.18, 0.47; k^2^ = 0.37, *p* = 0.002). The standardized indirect effect (EI → LMX → burnout) for *males*, as well, was −0.11 (95% CI: −0.23, −0.04; k^2^ = 0.14, *p* = 0.004). Table 4 summarizes the results.

**TABLE 4:** Hypotheses summary (Study 2).

Hypotheses	Male group	Female group
H2.1: LMX has a positive correlation with organizational justice	Supported	Supported
H2.2: LMX has a negative correlation with burnout	Supported	Supported
H2.3: LMX has a negative correlation with CWBs	Not supported	Supported
H2.4: EI has a positive correlation with LMX	Supported	Not supported
H2.5: EI has a positive correlation with organizational justice	Supported	Supported
H2.6: EI has a negative correlation with burnout	Supported	Supported
H2.7: EI has a negative correlation with CWBs	Not supported	Not supported
H2.8: LMX mediates the relationship between EI and organizational justice	Supported	Not supported
H2.9: LMX mediates the relationship between EI and burnout	Supported	Not supported
H2.10: LMX mediates the relationship between EI and CWBs	Not supported	Not supported
H2.11: Gender moderates the associations between EI, LMX, organizational justice, burnout, and CWBs	Supported	

## Study 3

In this study, we wanted to investigate if different motives corresponded with related attitudinal outcomes. We chose personal drivers (intrinsic/extrinsic motivation) as predictors of corresponding outcomes (intrinsic/extrinsic satisfaction) via LMX mediation. Intrinsic motivation is the internal driver for the individual’s experiences, which connect with self-concept and are inherently interesting or enjoyable. Thus, employees work out of the excitement, feeling of accomplishment, and personal satisfaction they derive from both the process of carrying out work-related activities and the results (Bauer et al., 2016; Legault, 2016). Extrinsic motivation is influenced by the organization, the work, and the employee’s environment (e.g., social norms, peer influence, financial needs, authority, or promises of reward), and it is focused on the utility of the activity rather than the activity itself (Legault, 2016).

### Intrinsic/Extrinsic Motivational Orientations

The intrinsic/extrinsic division of motivation lacks coherent research within the LMX paradigm. In addition, most of the past research on separate effects of intrinsic and extrinsic motivation has addressed the intrinsic aspect (e.g., Bauer et al., 2016). In addition, motivation has been shown to be affected by personal traits, needs, and even work fit, while affecting various outcomes and attitudes, such as satisfaction, OCBs, and engagement, making an understanding of intrinsic and extrinsic motivational orientations relevant to LMX mediation and gender moderation (e.g., Tziner et al., 2019b; Shkoler and Kimura, 2020).

### Intrinsic and Extrinsic Job Satisfaction

Job satisfaction is an internal state of gratification or discontentment about one’s job (Thompson and Phua, 2012). There can be distinctions between overall job satisfaction and subtypes of job satisfaction, such as intrinsic and extrinsic job satisfaction, and the latter may be more closely related to motivational states relative to global job satisfaction (Weiss et al., 1967). Satisfaction has been shown to be affected by *job experiences* (e.g., Mas-Machuca et al., 2016; Pacheco and Webber, 2016) and *individual demographical differences* (e.g., Pacheco and Webber, 2016; Shkoler and Kimura, 2020). Therefore, we suggest that women and men may have different levels of drivers/motives in their work and might enjoy/interpret intrinsic/extrinsic incentives differently. In practice, managers may supply the employee with various resources and incentives, such as rewards, work conditions (i.e., extrinsic incentives), and/or challenge, support, and work enjoyment (i.e., intrinsic incentives). In providing for the internal/external needs of the employee, managers may increase the different types of job satisfaction.

#### LMX and Job Satisfaction

Managers may provide their employees with various resources and incentives that are extrinsic, such as objective rewards and pleasant working conditions, or intrinsic, such as challenge, support, and work enjoyment. These incentives satisfy the internal and external needs of the employees whose various manifestations of job satisfaction are thereupon likely to be enhanced.

#### Motivation, LMX, and Job Satisfaction (LMX as Mediator)

In the same vein, given enough incentives, intrinsic/extrinsic motivation—understood as the expression of different drivers that move individuals to satisfy them—may also translate into intrinsic/extrinsic satisfaction. It appears that this outcome, by definition, necessitates a mediator between motivation and satisfaction. Indeed, we believe there to be an axis that connects the driver to the satisfaction and that given that the manager is a pinnacle in the work context (providing incentives and work-related resources), it is highly probable that LMX may act as a mediator in this regard.

#### Gender as a Moderator

Here, as well, we expect gender differences. Concerning Study 3, women and men may have different drivers/motives in their work and might enjoy/interpret intrinsic/extrinsic incentives differently.

All the proposed relationships described above are illustrated in Figure 4, and they give rise to the following hypotheses.

### Study 3 Hypotheses

H3.1: LMX has a positive correlation with intrinsic satisfaction.
H3.2: LMX has a positive correlation with extrinsic satisfaction.
H3.3: Intrinsic motivation has a positive correlation with intrinsic satisfaction.
H3.4: Intrinsic motivation has a positive correlation with extrinsic satisfaction.
H3.5: Extrinsic motivation has a positive correlation with intrinsic satisfaction.
H3.6: Extrinsic motivation has a positive correlation with extrinsic satisfaction.
H3.7: Intrinsic motivation has a positive correlation with LMX.
H3.8: Extrinsic motivation has a positive correlation with LMX.
H3.9: LMX mediates the relationship between intrinsic motivation and intrinsic satisfaction.
H3.10: LMX mediates the relationship between intrinsic motivation and extrinsic satisfaction.
H3.11: LMX mediates the relationship between extrinsic motivation and intrinsic satisfaction.
H3.12: LMX mediates the relationship between extrinsic motivation and extrinsic satisfaction.
H3.13: Gender moderates the associations between intrinsic/extrinsic motivation and intrinsic/extrinsic satisfaction (as depicted in H3.1–H3.12).

### Method

#### Procedure

The survey research (paper and pencil) was based on the administration of questionnaires by students who participated as research assistants (not as participants). The participation of the respondents in the survey was voluntary. We assured the anonymity and discretion of the participants and the data derived from the research and included a conscious consent question at the beginning of the survey asking for their agreement to participate. No incentives were given whatsoever to the participants for their cooperation (refer to the Ethics Statement section at the end of the paper).

#### Participants

Cross-sectional data were collected via voluntary surveys from 350 Israeli workers (all measures were self-reported) from various organizations, 38% males (*n* = 133) and 62% females (*n* = 217) aged between 20 and 67 (*M* = 27.06, *SD* = 6.62). Half of them (50%) had a high-school education, 39% held a BA degree, and 11% held an MA degree or higher.

#### Measures

*LMX* was gauged by the LMX7 questionnaire (Graen and Uhl-Bien, 1995) consisting of seven Likert-type items; however, each item had a different scale from 1 to 6. In the current research, reliability was α = 0.84 (*M* = 4.14, *SD* = 0.87).

*Motivation* was gauged by the Work Extrinsic and Intrinsic Motivation Scale (WEIMS; Tremblay et al., 2009) consisting of 18 Likert-type items between 1 (“does not correspond at all”) and 6 (“corresponds exactly”). The measure is (largely) divided into two subscales—*intrinsic* motivation (7 items) and *extrinsic* motivation (11 items). *Intrinsic motivation* had a reliability of α = 0.76 (*M* = 3.38, *SD* = 0.94, e.g., “For the satisfaction I experience from taking on interesting challenges”), and *extrinsic motivation* had a reliability of α = 0.70 (*M* = 3.97, *SD* = 1.15, e.g., “For the income it provides me”).

*Satisfaction* was gauged by the Minnesota Satisfaction Questionnaire—Short Form (MSQ-SF; Weiss et al., 1967) consisting of 20 Likert-type items between 1 (“very dissatisfied”) and 6 (“very satisfied”). The measure is divided into two subscales—intrinsic satisfaction (13 items) and extrinsic satisfaction (7 items), drawing upon Herzberg (1966). In the current research, *intrinsic satisfaction* had a reliability of α = 0.92 (*M* = 4.43, *SD* = 0.85, e.g., “The chance to do different things from time to time”), and *extrinsic satisfaction* had a reliability of α = 0.83 (*M* = 4.48, *SD* = 0.90, e.g., “My pay and the amount of work I do”).

### Results

#### Common-Method Bias

In order to assess the extent to which inter-correlations among the variables might be an artifact of CMV, we employed three tests: (a) the Harman’s single-factor method (a CFA in which all items are simultaneously loaded on one single factor), (b) a common latent factor method (a CFA in which all items are loaded on both their expected factors and one common latent factor is loaded on each of the items, respectively, but are uncorrelated to their respective latent factors), and (c) a CFA without a common latent factor, as suggested by Podsakoff et al. (2003) and advocated in Jawahar et al. (2018). The Harman’s single-factor method accounted only for 33.41% of the explained variance: χ^2^(311) = 977.31, *p* = 0.000, χ^2^/df = 3.14, CFI = 0.81, NFI = 0.82, GFI = 0.90, SRMR = 0.13, RMSEA (90% CI) = 0.17 (0.13–0.28), *p-close* = 0.005. In addition, the latent common method factor analysis accounted only for 31.55% of the explained variance: χ^2^(294) = 891.45, *p* = 0.000, χ^2^/df = 3.03, CFI = 0.88, NFI = 0.87, GFI = 0.95, SRMR = 0.09, RMSEA (90% CI) = 0.11 (0.07–0.16), *p-close* = 0.013. Last, the CFA analysis (without a common latent factor) accounted only for 32.81% of the explained variance: χ^2^(244) = 773.15, *p* = 0.000, χ^2^/df = 3.16, CFI = 0.73, NFI = 0.80, GFI = 0.89, SRMR = 0.11, RMSEA (90% CI) = 0.15 (0.11–0.27), *p-close* = 0.000. As can be seen, the common latent factor method produced better indices and less CMV. However, while these results do not completely rule out the possibility of same-source bias (i.e., CMV), according to Podsakoff et al. (2003), less than 50% (*R*^2^ < 0.50) of the explained variance accounted for by the first emerging factor indicates that CMV is an unlikely explanation of our investigation’s findings, in conjunction with the bad model fit for each analysis. In addition, we followed the suggestion for correcting CMV via construct-level correction made by Tehseen et al. (2017) and discovered that the changes in coefficients’ strength were very negligible. This, again, indicates that our results did not suffer from CMV issues.

**FIGURE 7:**
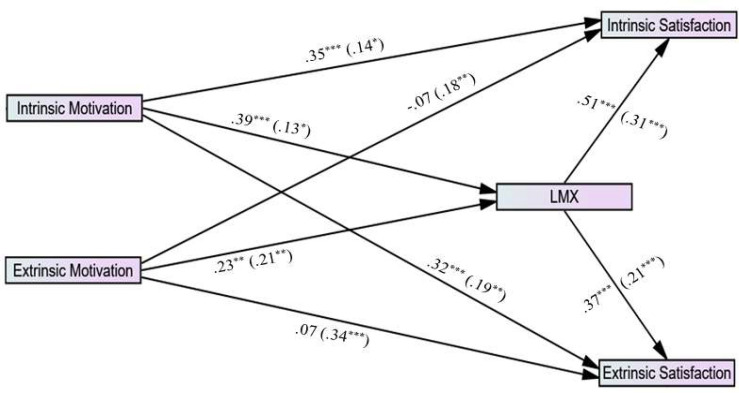
Path diagram for male group (*n* = 133) and female group (*n* = 217; in parenthesis), study 3. *p < 0.05, **p < 0.01, ***p < 0.001. χ^2^(df) = 5.17 (4), *p* = 0.461, χ^2^/df = 1.29, SRMR = 0.03, CFI = 0.94, GFI = 0.99, NFI = 0.95, ECVI = 0.21, RMSEA (90% CI) = 0.02 (0.00–0.07), *p*-close = 0.597.

In order to test the Study 3 model, we mainly employed SEM with multi-group moderation analyses using the observed (not latent) variables of the research. The path diagrams for the male group and the female group are presented in Figure 7, with the coefficients and significance levels (and fit indices). The bivariate correlation matrix is presented in Table 5.

**TABLE 5:** Correlation matrix for males (*n* = 133) and females (*n* = 217; *above* the diagonal) for Study 3.

	1	2	3	4	5	*M*_*m*_ (*M*_*f*_)	*SD*_*m*_ (*SD*_*f*_)
(1) I-M	–	0.39***	0.21**	0.27***	0.37***	3.75 (3.16)	0.80 (0.94)
(2) E-M	0.35***	–	0.26***	0.32***	0.47***	3.99 (3.96)	1.17 (1.13)
(3) LMX	0.47***	0.37***	–	0.38***	0.34***	4.10 (4.16)	0.97 (0.79)
(4) I-S	0.56***	0.24**	0.65***	–	0.89***	4.48 (4.40)	0.87 (0.83)
(5) E-S	0.52***	0.32***	0.54***	0.90***	–	4.53 (4.44)	0.85 (0.92)

**p < 0.01, ***p < 0.001. An indication of m or f: m, male group; f, female group. I-M, intrinsic motivation. E-M, extrinsic motivation. I-S, intrinsic satisfaction. E-S, extrinsic satisfaction.

As can be seen in Figure 7, the model’s fit is strong (see Byrne, 2010). We followed our analyses and processes as in Study 1. We used kappa-squared to test the indirect mediation effect with a 95% confidence interval bootstrapping.

This resulted in standardized indirect effects for *males*: (1) indirect effect of intrinsic motivation → LMX → intrinsic satisfaction of 0.25 (95% CI: 0.11, 0.30; k^2^ = 0.05, *p* = 0.000) and (2) an indirect effect of intrinsic motivation → LMX → extrinsic satisfaction of 0.19 (95% CI: 0.06, 0.25; k^2^ = 0.02, *p* = 0.000). In addition, the standardized indirect effects for *females*: (1) indirect effect of intrinsic motivation → LMX → intrinsic satisfaction of 0.07 (95% CI: 0.003, 0.11; of k^2^ = 0.01, *p* = 0.031), (2) indirect effect of intrinsic motivation → LMX → extrinsic satisfaction of 0.05 (95% CI: 0.002, 0.08; k^2^ = 0.01, *p* = 0.032), (3) indirect effect of extrinsic motivation → LMX → intrinsic satisfaction of 0.09 (95% CI: 0.01, 0.15; k^2^ = 0.12, *p* = 0.014), and (4) indirect effect of extrinsic motivation → LMX → extrinsic satisfaction of 0.06 (95% CI: 0.004, 0.12; k^2^ = 0.13, *p* = 0.000). The most notable results are the gender differences between the two models, namely: (1) extrinsic motivation does not work as hypothesized for males, based on an insignificant path from E-M to E-S; but (2) extrinsic motivation does work for females. Table 6 summarizes the results.

**TABLE 6:** Hypotheses summary (Study 3).

Hypotheses	Male group	Female group
H3.1: LMX has a positive correlation with intrinsic satisfaction	Supported	Supported
H3.2: LMX has a positive correlation with extrinsic satisfaction	Supported	Supported
H3.3: Intrinsic motivation has a positive correlation with intrinsic satisfaction	Supported	Supported
H3.4: Intrinsic motivation has a positive correlation with extrinsic satisfaction	Supported	Supported
H3.5: Extrinsic motivation has a positive correlation with intrinsic satisfaction	Not supported	Supported
H3.6: Extrinsic motivation has a positive correlation with extrinsic satisfaction	Not supported	Supported
H3.7: Intrinsic motivation has a positive correlation with LMX	Supported	Supported
H3.8: Extrinsic motivation has a positive correlation with LMX	Supported	Supported
H3.9: LMX mediates the relationship between intrinsic motivation and intrinsic satisfaction	Supported	Supported
H3.10: LMX mediates the relationship between intrinsic motivation and extrinsic satisfaction	Supported	Supported
H3.11: LMX mediates the relationship between extrinsic motivation and intrinsic satisfaction	Not supported	Supported
H3.12: LMX mediates the relationship between extrinsic motivation and extrinsic satisfaction	Not supported	Supported
H3.13: Gender moderates the associations between intrinsic/extrinsic motivation and intrinsic/extrinsic satisfaction	Supported	

## Discussion

The present research proposed to test leader–member relations (LMX) as a mediation mechanism, and gender as a related moderator, in various situations. Relatively, little research has been devoted to the prediction of LMX in terms of individual differences, and even less to the possible effects gender might have on the scope of LMX research. For this purpose, we proposed three different models and demonstrated that known, researched, and new relationships greatly depended on gender differences, as shown in each study.

### Study 1

As can be seen in Table 2, our predictions were supported only partially, mainly for the effect gender had on the results and the model in general. While LMX and individualism were positively correlated with OCBs in both genders, collectivism was only positively related to LMX and OCBs in the *male* group (in the female group, it was also significantly negatively related to LMX). As such, LMX acted as a *partial* mediator to OCBs from collectivism (male group only) and from individualism (female group only).

These findings support the current paper in a number of ways. First, IND/COL was tested in an organizational context and was shown to have positive associations with OCBs. Second, we verified that LMX might act as a partial mediator between individual differences (in terms of values) and organizational outcomes. Third, we have proven that gender has an intricate effect on the research model.

Specifically, we argue that our results suggest that women may not experience the effects of the COL-based CVOs on OCBs because women may generally show a higher level of OCBs compared to men: *t*(243) = 2.07, *p* = 0.039, Cohen’s *d* = 0.25. If women do show a higher base rate of OCBs relative to men, then the effect of the COL-based CVOs would be less significant. In contrast, a naturally low base rate of OCBs for men might be enhanced via a high COL-based CVOs for men. It is also interesting to observe the negative effect from IND-based CVOs to LMX for women, which may suggest that women high in IND-based CVOs would not see leader relationships as an important target for influence. The individualistic women in our sample tended to have lower LMX.

### Study 2

As can be seen in Table 4, the hypotheses were supported only partially, again, mainly for the effect gender had on the results and the model in general. LMX and EI were positively correlated to justice perceptions, and negatively to burnout in both genders. However, EI was not related to CWBs in any gender group, while LMX was negatively related to CWBs in the *female* group only.

Furthermore, EI was positively correlated with LMX in the *male* group only. Contrary to a vast literature showing that females scored higher in EI than males (e.g., Mandell and Pherwani, 2003), in Study 2, the male group (*M* = 4.53) scored higher than females (*M* = 4.41) on EI: *t*(241) = 2.07, *p* = 0.040, Cohen’s *d* = 0.21, which might explain the findings that LMX is a *partial* mediator between EI and justice perceptions or CWBs, for *males* only.

These findings support the current paper in several ways: (1) EI may have an impact on leader–member relations, justice perceptions, burnout, and CWBs; (2) LMX may have a positive impact on justice perceptions, burnout and CWBs; (3) LMX may act as a partial mediator between individual differences (in terms of traits) and personal job-related outcomes (justice perceptions and burnout); and (4) gender has an important impact on these results.

### Study 3

As can be seen in Table 6, most of the hypotheses were supported. Specifically, *all* the hypotheses were supported for the *female* group; however, extrinsic motivation was not at all correlated to any type of job satisfaction for the male group. Again, this shows the effect gender had on the results and the model in general. As such, LMX was found to be a *partial* mediator between work motivations (intrinsic/extrinsic) to job satisfactions (intrinsic/extrinsic), apart from the aforementioned discrepancy for the male group.

These findings support the current paper in several ways: (1) intrinsic and extrinsic motivations may be positively correlated with LMX and intrinsic and extrinsic satisfaction; (2) LMX may have a positive impact on both intrinsic and extrinsic satisfaction; (3) LMX may act as a partial mediator between individual differences (in terms of drivers) and personal job-related outcomes (intrinsic and extrinsic job satisfaction); and (4) gender has an impact on the results. Overall, it is clear that LMX serves as a component of intrinsic and extrinsic satisfaction, but this role of LMX is more pronounced for men.

This concludes the two main goals of the paper, namely, that (a) LMX may act as a mediator in an organizational context, and (b) gender *does* matter, making dramatic differences in results.

## Implications for Research and Practice

There are several important implications of the current research—statistical, practical, and theoretical. First, the differences in effect sizes across all three studies in this paper are a solid example for reporting effect sizes, as significance levels can show neither the strength of the effects nor their power. For instance, in Figure 5, the significance for the indirect effect for the *female* group is 0.013 with effect size (k^2^) of 0.21. However, in Figure 7, the first indirect effect for the *male* group is significant at 0.000 but with effect size (k^2^) of 0.05, which is drastically lower judging by Cohen’s (1988) benchmarks for the proportion of variance accounted for in one variable by another (e.g., *R*^2^, η^2^, etc.), defined as small, medium, and large effect sizes as 0.01, 0.09, and 0.25.

Second, we have consistent support for the LMX mediation with gender moderation paths articulated across all three studies within this paper and, thus, from a practical and organizational point of view, the findings in this paper are overall intricate and intriguing. To illustrate: (a) Study 1 showed that individualistic females tended to have lower-quality leader–member relations (negative correlation); (b) Study 2 showed that high LMX lowered the tendency of CWBs in males, but not in females; and (c) Study 3, using indirect effects modeled through LMX, showed that extrinsic motivation was not a significant driver in males, but it was for females, while intrinsic motivation was significant for both genders. Just as important as managerial skills is the consciousness that the manager may act as a mediator between the worker and various outcomes (see Figure 1) that puts a significant responsibility on managers when considering intersections of LMX and gender. In conjunction with our findings, we would recommend the selection of managers who can adjust and choose different approaches and leadership styles when managing a male subordinate vs. a female one or when encountering situations requiring more democratic and participative rather than directive leadership styles.

To extend this idea, we encourage managers and others involved in selection and assessment to consider the intersection of gender and leadership in more detail because there is no one specific type of leadership that is appropriate to all situations (Mumford and Manley, 2003).

Instead, there are different styles of leadership, which are differentially effective in different contexts and serve as different criteria for leaders (Cox et al., 2003). Across multiple studies, there is evidence for a tendency for women to be more democratic and participative in leadership style when compared to men (Eagly and Johnson, 1990), which seems to suggest that if other factors are assessed and considered equal, women may be particularly suitable for leadership roles demanding democratic and participative behaviors.

Beyond the important work of Eagly and Johnson (1990), more contemporary studies also indicate that gender differences in respect to leadership do exist, but that these differences are complex and point to an intersection of how particular gendered behaviors are differentially valued or are otherwise dominant in organizations (de la Rey, 2005; Eagly and Carli, 2012; Collins et al., 2014).

This finding essentially means that gender does affect the manifestation of different leadership styles to some extent, although reasons for this difference are not necessarily genetic (Eagly and Carli, 2012). One implication of this idea is that designers of organizational systems may suggest differences in how performance metrics for leadership are developed and communicated to men and women, and how these are potentially assessed across men and women differently based on the types of leadership required for specific roles. The use of such variable performance metrics could be employed both for purposes of leader development and performance management and for areas of assignment within organizations. For example, gender could potentially be used as one factor in a selection battery, when considering candidates for staffing leadership roles requiring an enhanced democratic and participative leadership style. Within this paper, this recommendation is consistent with the findings of Study 1, which would suggest that collectively oriented or allocentric values also be included with gender when LMX is a key criterion for leader performance.

Another key fact to consider, pertinent to leader selection and assessment, is that gender may influence the specific characteristics subordinates used to make judgments about desired leader behaviors, and these judgments can include nuanced effects within LMX dimensions. For example, communally oriented LMX dimensions such as loyalty have been shown to influence job embeddedness for female (but not male) subordinates (Collins et al., 2014). In contrast, agentically oriented LMX dimensions such as respect have been shown to influence job embeddedness for both genders in similar ways (Collins et al., 2014). In this paper, we have shown a similar effect, in that LMX lowered the tendency of CWBs in males, but not in females. While CWBs are not the same as job embeddedness, they certainly share a negative association (Holtom and Darabi, 2018). We suggest that the finding of this paper that LMX lowered the tendency of CWBs in males should be tested in organizations with specific regard to agentically oriented LMX dimensions, to see if the effect of lowered CWBs could be enhanced.

In addition, in Study 3, we showed that extrinsic motivation was not a significant driver of satisfaction in males, but that it was for females—although intrinsic motivation was significant for both genders. This has implications for selecting female leaders into contexts heavy with extrinsic goal affordances, in developing performance management programs, and when discussing the types of motivational states potentially activated by goal setting and resourcing activities. For example, managers might consider the differential effect on job embeddedness from communally oriented LMX dimensions (such as loyalty) when selecting women as part of leader–subordinate dyads and when conducting performance management programs.

Additionally, the interaction of gender, LMX, and job embeddedness might be extended to other outcomes and downstream workplace processes related to LMX such as justice perceptions. Finally, we note that the notion of demographical differences—specifically gender—is underestimated in the literature on individual differences and leadership (Zagenczyk et al., 2015). However, as found in this paper, gender has an impact on research in various ways and should *not* be ruled out when investigating or replicating models for the selection of leaders. Its effects are important on theoretical and practical levels alike.

## Limitations

The use of self-report measures may prove a limitation, as may the cross-sectional data we collected in each study that resulted in a lack of inferences of causation. We also did not gauge the LMX from the manager/leader perspective, only from that of the employee. This brings up the related limitation that we did not employ dyadic match and directional match as it relates to gender in leader–subordinate pairs. However, Spector (2019) argues that longitudinal designs offer limited advantages over cross-sectional designs. The latter incorporates explanatory mechanisms and temporal precedence (e.g., gender) factors and constitutes a valuable mode of investigation (for further reading, see Spector, 2019).

A more serious limitation is that the fit indices we produced in Study 1 showed only a marginal fit, with RMSEA above 0.10. However, this was probably due to the measures chosen for IND and COL-based CVOs. Other measures of CVOs, such as the horizontal and vertical individualism and collectivism scale (HVIC; Triandis and Gelfand, 1998), could provide higher reliability and more nuanced distinctions of these CVOs. On a separate ground, one may question to what extent current results in regard to culture dimensions can extrapolate to other cultures if it is postulated that Israeli culture is quite unique (as a reminder, the measures in the current study were tapped with Israeli respondents). We posit that Almagor and Ben-Porath have demonstrated that results obtained in the Israeli cultural context replicate into the North American one. Most likely, because despite some uniqueness characterizing the Israeli culture, as any other culture, Sagie and Weisberg (2001) assert that Israeli society has gone from being ascetic, collectivistic, closed, and relatively homogeneous to being more materialistic, individualistic, open, and pluralistic as the North American is. Hence, we assume that current results in regard to the two investigated culture dimensions can be extrapolated to other cultural contexts. Nonetheless, we recommend to attempt replicate present findings also with respondents from other national cultures.

Another limitation might be the segmentation of the research into three studies. However, it would be near impossible to deliver such a demanding, long, and tiring survey containing all the research variables to one set of subjects. It may have been possible to logically combine the individual differences used in Study 1 and Study 2 into a single SEM. However, we were concerned about the relationships between emotional intelligence and collectivism items being too close, in that the items could have been related to both constructs to some degree. This also raises the issue of the inclusion of emotional intelligence as a construct.

The use of EI within this study, at least within the framework of its traditional interpretation, may be seen as a limitation, based on a new understanding of the general factor of personality (GFP). Significant evidence now exists for a degree of variability overlap between EI and the GFP that accounts for almost the entire construct (van der Linden et al., 2018). Meta-analytic evidence in particular points toward an estimate of ρ = 0.88 between EI and GFP (van der Linden et al., 2017), with further genetic studies suggesting a phenotypic correlation of *r* = 0.90 (van der Linden et al., 2018). There is some debate regarding the nature of the GFP, in respect to whether it is a substantive factor or rather it is a result of systemic statistical bias; however, the consensus seems to be that although there may be some amount of systemic bias, there remains a stable and substantive individual difference behind the GFP (van der Linden et al., 2018). As the GFP seems to represent the most desirable elements of the five-factor model of personality such as emotional stability, conscientiousness, and sociability, it seems to present a broad trait reflecting social effectiveness, which is quite similar to what EI is measuring (van der Linden et al., 2016). However, EI is an established construct, and links between EI and the GFP would be beyond the scope of this paper.

## Recommendations

In respect to the relationships between individual differences and LMX, one important point for future research would be to investigate data addressing the congruence of supervisor and subordinate individual difference in greater detail. Accordingly, we would recommend including gender or other demographics in future models/studies as they might have interesting and important roles based on their congruence or lack of congruence within supervisor and subordinate dyads, using effect sizes for mediating analyses and indirect effects (see Preacher and Kelley, 2011), testing LMX as a mediator in different model constellations (for example, by combining IND and COL-based CVOs with EI as antecedents to LMX in similar moderated-mediation models), and replicating the findings of the paper to reach better validity. In this regard, we would recommend more nuanced measures of IND and COL-based CVOs (e.g., the HVIC; Triandis and Gelfand, 1998). Finally, we recommend testing models with both job-related and individual differences parameters in an organizational context, with attention given to specific types of contextual effects (e.g., culture and climate), and highlight the vital role of the manager (especially via manager’s impressions of LMX) and the sensitivity one may need in managerial skills.

One of the main roles of academia, and ours as researchers, is to make advances in science and push it forward. Our focus within this paper was to examine the role of the moderated-mediation pathways that link LMX mediation and gender-based moderation, which serve as the link between the different studies we present in this paper. In this regard, we found consistent support that gender may have a moderating effect in conjunction with LMX. We believe this is because LMX encapsulates the strength of the relationship between leaders and subordinates.

We have also suggested that the results of this paper have a major implication for selection of women to leadership roles, in that we have articulated some of the contextual elements that allow women to emerge as effective leaders, although we note that the relationships between gender, leadership, and organizational outcomes are vast and complex. We consequently wish to call for more research on this issue in order to reach what is “necessary for a coherent science” (Reeve, 2016, p. 1).

## Data Availability Statement

The datasets generated for this study are available on request to the corresponding author.

## Ethics Statement

Ethical review and approval was not required for the study on human participants in accordance with the local legislation and institutional requirements. The patients/participants provided their written informed consent to participate in this study.

## Author Contributions

AT: study design and project management. OS: data analyses and manuscript writing. EF: manuscript writing. All authors contributed to the article and approved the submitted version.

## Conflict of Interest

The authors declare that the research was conducted in the absence of any commercial or financial relationships that could be construed as a potential conflict of interest.
